# Dysregulation of the Renin-Angiotensin System and the Vasopressinergic System Interactions in Cardiovascular Disorders

**DOI:** 10.1007/s11906-018-0823-9

**Published:** 2018-03-19

**Authors:** Ewa Szczepanska-Sadowska, Katarzyna Czarzasta, Agnieszka Cudnoch-Jedrzejewska

**Affiliations:** Department of Experimental and Clinical Physiology, Laboratory of Centre for Preclinical Research, Medical University of Warsaw, Banacha 1b, 02-097 Warsaw, Poland

**Keywords:** Aldosterone, Angiotensin receptors, Cardiovascular reflexes, Heart failure, Hypertension, Hypoxia, Renal failure, Vasopressin receptors

## Abstract

**Purpose of Review:**

In many instances, the renin-angiotensin system (RAS) and the vasopressinergic system (VPS) are jointly activated by the same stimuli and engaged in the regulation of the same processes.

**Recent Findings:**

Angiotensin II (Ang II) and arginine vasopressin (AVP), which are the main active compounds of the RAS and the VPS, interact at several levels. Firstly, Ang II, acting on AT1 receptors (AT1R), plays a significant role in the release of AVP from vasopressinergic neurons and AVP, stimulating V1a receptors (V1aR), regulates the release of renin in the kidney. Secondly, Ang II and AVP, acting on AT1R and V1aR, respectively, exert vasoconstriction, increase cardiac contractility, stimulate the sympathoadrenal system, and elevate blood pressure. At the same time, they act antagonistically in the regulation of blood pressure by baroreflex. Thirdly, the cooperative action of Ang II acting on AT1R and AVP stimulating both V1aR and V2 receptors in the kidney is necessary for the appropriate regulation of renal blood flow and the efficient resorption of sodium and water. Furthermore, both peptides enhance the release of aldosterone and potentiate its action in the renal tubules.

**Summary:**

In this review, we (1) point attention to the role of the cooperative action of Ang II and AVP for the regulation of blood pressure and the water-electrolyte balance under physiological conditions, (2) present the subcellular mechanisms underlying interactions of these two peptides, and (3) provide evidence that dysregulation of the cooperative action of Ang II and AVP significantly contributes to the development of disturbances in the regulation of blood pressure and the water-electrolyte balance in cardiovascular diseases.

## Introduction

Angiotensin II (Ang II) is a member of the renin-angiotensin system (RAS), which forms a cascade of highly active biological compounds regulating a variety of physiological functions. Co-activation of the RAS and the vasopressinergic system (VPS) by the same stimuli, and engagement of the same post-receptor intracellular pathways by Ang II and vasopressin (AVP), favors formation of different types of interactions, which may result in additive, synergistic, or antagonistic effects. Both Ang II and AVP directly participate in the regulation of the cardiovascular system. They also have an impact on several other processes, such as metabolism, stress, emotional disorders, and inflammation, which may exert potent secondary effects on the cardiovascular functions. The main purpose of the present review is to draw attention to the interactions of Ang II and AVP in the regulation of the water-electrolyte balance and blood pressure in healthy individuals and in patients with cardiovascular diseases. Specifically, we give a description of the regulation and function of the RAS and the VPS and we focus on the mechanisms underlying the interactions of Ang II and AVP in cardiovascular regulation and on disturbances of these interactions in cardiovascular diseases.

## Renin-Angiotensin System

Prorenin and renin circulating in the blood are produced mainly in the juxtaglomerular epithelioid (JGE) cells, located in the wall of the afferent arterioles at the entrance to the glomeruli and in close vicinity to the macula densa (MD) cells, and forming together with the MD cells the juxtaglomerular apparatus (JGA) [1]. Renin is an aspartyl protease encoded by a single gene located in chromosome 1q42. During posttranslational processes, preprorenin, which consists of 401 amino acid residues, is converted to prorenin and subsequently to renin [2, 3]. Renin is a highly specific enzyme, which acts on renin substrate—angiotensinogen (Agt)—and catalyzes the first step in a cascade of RAS compounds. Acting on Agt, renin cleaves a decapeptide—angiotensin I (Ang I) which can be converted to Ang II by angiotensin-converting enzyme 1 (ACE, ACE 1) or to angiotensin-(1-7) [Ang-(1-7)] by ACE 2 and other enzymes. ACE 1 is a glycoprotein with a molecular mass of 18 kDa and two active carboxy-terminal sites, which can also metabolize bradykinin (BK), an active vasodilator and natriuretic substance, to an inactive metabolite [4, 5]. ACE 2 is a zinc metalloproteinase consisting of 805 amino acids, showing significant homology to ACE 1; however, the enzymatic activity of ACE 2 is not inhibited by ACE 1 inhibitors. ACE 2 hydrolyzes Ang I to Ang-(1-9), and Ang II to Ang-(1-7) [6].

Other angiotensin peptides [Ang-(1-9), Ang III (Ang-(2-8)), Ang IV (Ang-(3-8), Ang-(1-4), Ang-(1-5), Ang-(5-8) and Ang-(1-12)] are generated by aminopeptidases, carboxypeptidases, endopeptidases, and chymase. Prorenin, renin, and angiotensins act by means of specific renin (PRR) and angiotensin receptors, which are both widely distributed in the cardiovascular system and other organs (see below).

### Local Renin-Angiotensin Systems

#### Renal RAS

All components of the RAS are present in the kidney [7, 8]. Renin is released from the JGA cells into the blood, but some small portion of the JGA renin is filtered through the glomeruli and reabsorbed in the proximal tubules [9]. There is also evidence for the synthesis of renin by the principal cells of the collecting ducts [10].

The synthesis and release of renin is altered by factors regulating the production of cyclic adenosine monophosphate (cAMP) and cyclic guanosine monophosphate (cGMP) and by changes in the intracellular calcium and sodium content. The stimulatory cAMP-mediated effect of β-adrenergic stimulation on release of renin from the JGA cells plays a significant role during the activation of the sympathetic nervous system [11]. Prostanoids, which stimulate renin secretion via activation of prostaglandin E2 (EP2), prostaglandin E4 (EP4), and prostacyclin (IP) receptors, also act via a Gs-mediated increase in cAMP. Low level of calcium in the extracellular space and in the JGA cells exerts an intensifying effect on the release of renin, through the phenomenon known as the “calcium paradox” [12]. It has been postulated that the inhibitory effect of elevated calcium level is caused by an inhibition of renin gene transcription and destabilization of renin mRNA [13]. Nitric oxide (NO) and atrial natriuretic peptides are involved in the stimulation of renin release through a cGMP-dependent pathway [14, 15]. The release of renin is inhibited by an increase in the intraglomerular hydrostatic pressure and this effect depends on the stimulation of the adenosine A1 receptors. Experimental and clinical studies have provided evidence for the inhibition of renin secretion by a high sodium intake [16, 17]. Besides, Ang II inhibits renin release directly in a short negative feedback loop by means of the AT1 receptors (AT1R), and indirectly through a suppression of prostanoid synthesis induced by cyclooxygenase-2 (COX-2) [18•]. The role of AVP in the regulation of renin release is more complex as AVP stimulates the release by acting on the V2 receptors (V2R) in the JGA cells. However, it can also inhibit the release of renin when it acts on the V1a receptors (V1aR) in the macula densa cells [19]. In hypertensive concentrations, both Ang II and AVP exert indirect inhibitory effects, secondary to the increases in the systemic and intraglomerular blood pressure. Acting centrally, Ang II and AVP may both either stimulate or inhibit the release of renin via activation of specific groups of cardiovascular neurons in the brain (see below). Aldosterone and glucocorticoids stimulate the synthesis of renin in the JGA cells, however, both of these steroids can also exert several indirect effects resulting from their actions on sodium and the water balance and synthesis of other regulatory compounds. These actions are particularly important in salt-sensitive hypertension [20•].

#### Cardiovascular RAS

Renin, Agt, ACE, and Ang II receptors are all present in the myocardium. Angiotensinogen is present mainly in the cardiac atria and fibers of the conductive system. The strongest expression of ACE was found in the coronary endothelial cells and cardiac fibroblasts, and weaker expression of ACE was found in the aorta, pulmonary arteries, valves, endocardium, and epicardium [21–23]. Angiotensin I and Ang II are synthesized de novo in the heart and their synthesis is regulated by glucocorticoids, estrogen, and thyroid hormones [24]. In the rat, endothelial cell ACE gene expression is stimulated by aldosterone [25]. Stimulation of beta receptors upregulates renin and Agt expression in the heart and mechanical stretching of the ventricular myocytes increases the release of Ang II [21, 26].

#### Brain RAS

All components of the RAS have been detected in the brain, in particular, in the brain cardiovascular regions, including the paraventricular nucleus, the medullary cardiovascular regions, and the circumventricular organs. There is strong evidence that the intrabrain RAS plays a significant regulatory role in cardiovascular regulation and is significantly affected in cardiovascular diseases [27–30]. For example, it has been shown that the intracerebroventricular (ICV) administration of renin antisense into the brain reduces blood pressure in spontaneously hypertensive rats (SHR) (see also below) [31, 32]. It has also been shown that hyperosmotic stimulation increases the release of Ang II in the paraventricular nucleus [33] which suggests that it may play a role under physiological conditions.

#### Adrenal Gland RAS

The components of the RAS have been identified in the adrenal medullary chromaffin cells [34]. Significant renin activity was detected in the zona glomerulosa of the adrenal cortex and smaller expressions were present in the zona fasciculata and reticularis [35]. Sodium restriction or a high potassium diet increased renin concentration and aldosterone synthesis in the adrenal gland [36]. It was also shown that stimulation of aldosterone secretion by potassium or adrenocorticotropic hormone (ACTH) involves activation of ACE and AT1R [37].

### Cellular Effects of Angiotensins

#### AT1 Receptors

The human angiotensin AT1 receptor gene (*at1r*) has been mapped to chromosome 3 [38]. The AT1R protein is composed of 359 amino acids and belongs to G protein-coupled receptors (GPCR) [39, 40]. After interaction with Ang II, the AT1Rs are internalized but approximately 25% of the internalized receptors recycle back to plasma membrane. The remaining AT1R serve their intracellular functions or are degraded in lysosomes [41]. The ACE→Ang II→AT1R axis forms the vasoconstrictor/proliferation/profibrotic wing of the RAS. Angiotensin AT1Rs are widely distributed in the heart and vessels, kidney, brain, lungs, endocrine glands, and several other organs [42, 43]. Stimulation of AT1Rs by Ang II involves activation of a broad scope of intracellular pathways, which may have different roles in specific groups of cells [44]. With respect to the regulation of the cardiovascular system and Ang II and AVP interactions, the most essential is the activation of phospholipase C (PLC) and phospholipase D (PLD) with subsequent formation of inositol triphosphate (IP3) and diacylglycerol (DAG). Increased production of IP3 results in the release of calcium from intracellular stores and subsequent activation of Ca^2+^-dependent myosin light-chain kinase (MLCK) [45]. Activation of PLD causes hydrolysis of phosphatidylcholine and production of phosphatidic acid, which can be transformed into diacylglycerol, an activator of conventional and novel classes of protein kinase of type C (PKC) [46•].

Activation of AT1R also results in the stimulation of phospholipase A2 (PLA2) and the production of arachidonic acid. Metabolism of arachidonic acid by cyclooxygenases, lipoxygenases and cytochrome P450 enzymes results in the formation of eicosanoids which regulate the contractility of the vascular smooth muscle cells (VSMC) [47]. The main signaling pathways activated by AT1R stimulation involve nicotinamide adenine dinucleotide phosphate (NADPH) oxidase, metalloproteinases (MMP), and the peroxisome proliferator-activated receptors (PPAR). The stimulation of AT1R results in the activation of NADPH oxidase and the subsequent generation of reactive oxygen species (ROS). This promotes the formation of several molecules engaged in inflammatory and profibrotic processes. Among them are mitogen-activated protein kinases [p38 MAPK, c-Jun N-terminal kinases (JNK), extracellular signal-regulated protein kinases 1 and 2 (ERK1/2), extracellular-signal-regulated kinase 5 (ERK5)] and transcription factors [nuclear factor kappa-light-chain-enhancer of activated B cells (NF-κB), activator protein 1 (AP-1), hypoxia-inducible factor 1-alpha (HIF-1α)]. There is also evidence that the stimulation of AT1R is associated with increased activity of Na^+^,K^+^-ATPase (NKA). The activation of AT1R also results in the downregulation of PPAR, which acts as an anti-inflammatory link [46•, 48, 49]. The activation of ERK1/2, p38MAPK, and c-Jun kinase pathways mediates the migratory and pro-proliferative actions of AT1R whereas an increase of interstitial matrix MMP activity and reduced production of tissue inhibitor of MMP-1 contribute to increased collagen accumulation. To some extent, the activation of ERK1/2 and p38 depends on the transactivation of epidermal growth factor receptor (EGFR) and the stimulation of proteins of the Src family of tyrosine kinase by a mechanism involving MMP [50]. The process of transactivation critically depends upon agonist binding by β-arrestin2 [51]. AT1R can also be activated by mechanical stretching through the mechanism independent of Ang II [52, 53•].

Homologous regulation of AT1 receptors by Ang II appears to depend on the type of cells. In a culture of vascular smooth muscle cells, Ang II downregulated AT1R mRNA and protein expression [54], whereas Ang II increased the expression of AT1R in neurons and the effect was associated with elevated expressions of NF-κB and Ets-like protein (Elk-1) [55••]. In renal mesangial cells, stimulation of AT1R by Ang II stimulated stress activated protein kinase (SAPK) through the mechanism of engaging stimulation of pertussis toxin-sensitive and pertussis toxin-insensitive G proteins and activation of tyrosine kinase [56]. Significant upregulation of AT1R mRNA was found in the rat subfornical organs (SFO) and anterior pituitary after sustained (5 days) dehydration [57]. In healthy human subjects, prolonged (8 days) blockade of AT1R increased PRA and Ang II concentrations, elevated urinary sodium excretion, decreased plasma aldosterone concentration, and reduced filtration fraction (FF) [58].

There is substantial evidence that AT1Rs are overexpressed in cardiovascular diseases. Expression of AT1R is elevated in SHR arterial vessels, in the brain and hearts of rats with 2K-1C hypertension, and in various brain regions [paraventricular nucleus (PVN), rostral ventrolateral medulla (RVLM), SFO] of hypertensive or myocardially infarcted rats and of rats exposed to oxidative stress or drastic acute and mild chronic stress [29, 59–65]. There is evidence that AT1Rs are upregulated by aldosterone, endothelin, and tumor necrosis factor alpha (TNF-α) [61, 66] and downregulated by Ang-(1-7), NO, high-density lipoprotein (HDL) cholesterol, and stimulation of AT2R [67–69]. In neuronal cultures prepared from the brains of SHR and Wistar Kyoto (WKY) rats, the expression of AT1R was significantly greater in SHR than in WKY and in both strains it was upregulated by elevated sodium concentration [70]. Several studies have provided evidence that the upregulation of AT1R or other compounds of the RAS may have functional consequences in cardiovascular pathology. For example, overactivation of AT1R in the RVLM plays an important role in the overstimulation of the sympathetic activity in stroke prone SHR [71•]. A significant reduction of blood pressure and a decreased release of Ang II in the hypothalamus was found in chronic 2K-1C hypertensive rats after antisense inhibition of the synthesis of Agt in the brain [72], and deficiency in Agt expression in the brain of Agt(−) transgenic rats was associated with reduced sympathoexcitation and pressor responses to ouabain [73]. Moreover, intracardiac pretreatment with AT1R antisense prevented the development of hypertension and cardiac hypertrophy and reduced cardiovascular contractility in SHR exposed to chronic infusion of Ang II [74]. Oral administration of AT1R antagonists reduced hypertension and cerebral edema in stroke-prone SHR, and oral administration of captopril decreased the resting blood pressure and cardiovascular responses to stress in myocardially infarcted rats [75•, 76, 77]. Furthermore, the *Macaca fascicularis* monkey maintained on a high cholesterol diet responded to prolonged administration of losartan with a significant reduction of the size of atherosclerotic fatty streaks in the aorta, coronary arteries, and carotid artery [78]. Similarly, blockade of AT1Rs with telmisartan reduced the size of the atherosclerotic damage and superoxide production in apolipoprotein E (ApoE)-deficient mice [79].

#### AT2 Receptors

The gene for AT2 receptor (AT2R) is located on the X chromosome [80]. The AT2R protein belongs to a family of G protein-coupled proteins and has a low homology of amino acid sequence (~ 34%) with AT1R [81]. Stimulation of AT2R activates phosphotyrosine phosphatases, especially serine/threonine phosphatase 2A, protein kinase phosphatase, and SHP-1 tyrosine phosphatase. This is associated with an inactivation of MAPK (specifically p42 and p44 MAPK) and ERK [82]. The most prominent expression of AT2R was found in the kidney, heart, blood vessels, and brain, especially in the soma and dendrites of the PVN [83•]. During cold-restraint stress, expression of AT2R in the brain increases after blockade of AT1R [84]. In the kidney, the AT2Rs are present in the glomerular epithelial cells, cortical tubules, and interstitial cells [85]. In the heart, AT2Rs are present in the atrial and ventricular myocardium and in the vascular smooth muscle cells of the coronary arteries [86]. Expression of AT2R is upregulated by sodium depletion and by insulin and insulin-like growth factor 1 (IGF-1). It is inhibited by Ang II and growth factors such as platelet-derived growth factor (PDGF) and epidermal growth factor (EGF) [85, 87, 88].

In the cardiovascular system, activation of AT2R exerts opposite effects to those following stimulation of AT1R. After blockade of AT1R, administration of Ang II may result in hypotension, partly mediated by an increased production of BK, NO, and cGMP [89]. In addition, stimulation of AT2R exerts antiproliferative and proapoptotic effects on smooth muscle cells, and reduces expression of AT1R and transforming growth factor beta (TGF-β) receptors [90]. In the heart, stimulation of the AT2Rs inhibits growth and remodeling and induces coronary vasodilation [91]. The role of AT2R increases under pathological conditions. They play a buffering role by preventing cardiac hypertrophy and fibrosis during the administration of Ang II and participate in cardiac remodeling during post myocardial infarction. It is also postulated that they act nephroprotectively in chronic kidney diseases [92].

#### Angiotensin-(1-7) Receptors

Ang-(1-7) is the main non-classical RAS peptide, which exerts actions via the G protein-coupled receptor Mas [93]. In many aspects, stimulation of Mas receptors opposes the negative effects of stimulation of AT1R and has similar functional consequences as the stimulation of AT2R. It has been found that stimulation of the ACE2→Ang-(1-7)→Mas receptor axis exerts significant vasodepressor and antihypertensive actions. These actions can be unmasked after the inhibition of ACE or AT1R. Ang-(1-7) can also act indirectly through the stimulation of the release of prostaglandins (PG) and NO and via interaction with bradykinin [94, 95]. Ang-(1-7) inhibits the growth of cardiomyocytes and smooth muscle cells, reduces the activity of MAP kinase, inhibits the generation of ROS, reduces Ang II-induced stimulation of ERK1/2 and Rho kinases, causes the downregulation of AT1Rs, and attenuates the unfavorable structural and functional consequences of post-infarct heart failure and cerebral ischemia [67, 93, 96–98]. In the kidney, Ang-(1-7) counteracts the stimulatory effect of Ang II on Na^+^,K^+^-ATPase and promotes water transport in the rat inner medullary collecting duct. The latter effect can be abolished both by Mas and V2R antagonists, which suggests involvement of AVP in this process [99, 100]. Stimulation of Mas receptors reduces symptoms of experimentally evoked glomerulosclerosis, namely, decreases expression of TGF-β, plasminogen activator inhibitor-1 (PAI-1), fibronectin (FN), and collagen I [101].

Current evidence indicates that Ang-(1-7) acting in the brain may exert both depressor and pressor effects depending on the locus of action. Presumably, some of the pressor effects are mediated by AT1 receptors or by AVP, which is released by Ang-(1-7) [102–104]. It was found that administration of Ang-(1-7), either ICV or into the nucleus of the solitary tract (NTS), induces hypotensive and bradycardic effects and facilitates baroreflex bradycardia, whereas the intrabrain administration of Ang-(1-7) antibody in transgenic hypertensive rats induces tachycardia and hypertension [105, 106•]. Moreover, selective overexpression of ACE 2 in the brain prevented development of neurogenic hypertension in mice through improvement of spontaneous baroreflex sensitivity and parasympathetic activity [107•]. In contrast, blockade of Mas receptors in the PVN resulted in a reduction of the renal sympathetic nerve activity (RSNA), which may suggest involvement of Ang-(1-7) in tonic maintenance of RSNA by the PVN [108]. Administration of Ang-(1-7) antagonist accelerated and intensified development of hypertension in the two-kidney, one-clip Goldblatt model [109•] and Mas knockout mice manifested reduced diuresis and natriuresis, and this was associated with glomerular hyperfiltration and increase of collagen III and fibronectin content in the mesangium and interstitium. They also showed upregulation of AT1R and TGF-β mRNA expression [110]. Altogether, the above results indicate that Ang-(1-7) may protect the cardiovascular system to some extent from destructive changes induced by Ang II.

#### Other Angiotensin Receptors

Blood pressure and renal functions are also regulated by Ang III (Ang 4-8) and Ang IV (Ang 3-8) [111]. Angiotensin III is formed from Ang II via aminopeptidase A and acts via stimulation of the AT1 receptors [111]. Angiotensin IV has its own receptor—the transmembrane enzyme, insulin-regulated aminopeptidase (IRAP, AT4R), which is present in the brain, heart, blood vessels, and kidney. Some of the actions of Ang IV can be mediated by AT1R [112]. Expression of AT4R in the carotid body is significantly elevated by hypoxia [113].

### Role of the Renin-Angiotensin System in Cardiovascular Regulation

The renin-angiotensin system regulates blood pressure through actions exerted either directly on the cardiovascular system or indirectly by means of the autonomic nervous system and vasoactive endocrine factors affecting blood circulation and the water-electrolyte balance.

#### Direct Vascular Effects of Angiotensins

Several studies have shown that systemic and locally released Ang II plays a key role in the regulation of vascular tone and that overactivation of the RAS causes excessive vasoconstriction and vascular remodeling, fibrosis, and atherosclerosis of the vascular wall resulting from endothelial dysfunction, as well as migratory, growth promoting, proliferative, apoptotic, and inflammatory processes [49, 114]. The vasoconstriction is caused by activation of AT1R conjugated to Gq/11 and G12/13 classes of G proteins [115, 116]. The vasoconstrictory effect of Ang II can only be partly reduced by the intracellular effect of Ang II on the potassium current (Kv), which is associated with hyperpolarization of smooth muscle cells [117]. In the vessels, heart, and kidney, Ang II promotes proinflammatory processes through induction of NF-κB and upregulation of cytokines and chemokines [interleukin 6 (IL-6), TNF-α, monocyte chemoattractant protein 1 (MCP-1)] which is followed by an increased expression of P-selectin, E-selectin, intercellular cell adhesion molecule-1 (ICAM-1), and vascular cell adhesion molecule-1 (VCAM-1) [118, 119]. Increased shear stress, which enhances ACE and basic fibroblast growth factor (bFGF) expression in the vascular smooth muscle cells, may additionally promote the negative effects of RAS overactivation [120].

Cardiovascular diseases are associated with inappropriate activation of the RAS in the vascular wall. For example, SHRs show greater expression of AT1R and lower expression of AT2R in mesangial vascular cells than WKY rats. Furthermore, ACE 2 gene transfer into SHR results in reduced perivascular fibrosis and blockade of AT1R inhibits Ang II-induced NF-κB activation and VCAM-1 accumulation in smooth muscle cells [119, 121].

#### Direct Cardiac Effects of Angiotensins

Angiotensin II, its precursors and cleavages are produced in the heart and distributed heterogeneously in the cardiac walls, endocardium, epicardium, conductive system, valves, aorta, pulmonary arteries, and coronary vessels [21, 23, 122]. Expression of elements of the RAS is higher in the fetal developing heart and in cardiac pathology than in the healthy heart of adults [23, 122]. Cardiac AT1Rs are coupled with Gαq and use ERK1/2 and phosphatidylinositol 3-kinase (PI3K) pathways and protein kinase B (Akt) phosphorylation as the main transduction cascades in Ang II-mediated cell proliferation. By stimulating AT1R, Ang II may also interact with G_αi_, and may regulate adenylyl cyclase and the unconventional arrestin beta 2 pathway as well as heterotrimeric signaling processes [123]. Systemic administration of Ang II evokes positive chronotropic and inotropic effects, modifies cardiac metabolism, and induces vasoconstriction of coronary blood vessels [124]. Exposure of cardiac fibroblasts to elevated Ang II concentration or overexpression of AT1R increases expression of focal adhesion kinases (FAK) and integrins in cultured neonatal cells, and elevates expression of c-fos, EGFR1, TGF-β, and extracellular matrix proteins in the cardiomyocytes of adults [125•, 126]. The above effects can be abolished by PKC inhibitors or EGF receptor kinase antagonists [127]. Renin and other components of the RAS play a role in the regulation of cell volume and chemical communication in the heart, for example, renin in cooperation with Agt impairs permeability of gap junctions [128•]. Moreover, it has been shown that Ang II, acting in the heart, increases the generation of hydroxyl radicals and impairs cell-to-cell communication, impulse propagation, and conduction velocity [129•, 130•]. Mechanical stretch stimulates secretion of Ang II from myocytes and increases Agt gene expression in cardiac myocytes and fibroblasts [21]. It is suggested that the cardiac AT1R functions as a mechanosensor and that the pressure overload may induce cardiac remodeling through mechanic activation of AT1R, independently of Ang II [53•]. Rats with hypertension induced by coarctation of the abdominal aorta show significant overexpression of AT1R mRNA and TGF-β mRNA, which are associated with cardiac hypertrophy. The latter two effects could be abolished by blockade of AT1Rs [131]. Studies on rats or mice with deletion or overexpression of various components of the RAS and on SHR provide additional arguments for the negative effects of the overexpression of Ang II in hypertension and post-infarct heart failure [132, 133]. In the samples of the left ventricle obtained from patients with post-infarct heart failure, the expression of AT1R was markedly elevated in patients with acute myocardial infarction and reduced in those with an old infarction or dilated cardiomyopathy. Expression of AT2R in the failing hearts was upregulated and this effect was particularly well seen in the fibrous regions of the myocardium [134].

#### Central Cardiovascular Effects of Angiotensins

The first evidence that Ang II induces a central pressor effect appeared over 50 years ago [135] and was confirmed in multiple studies [136, 137•, 138]. The pressor response may be evoked by intracerebroventricular (ICV) administration of Ang II or Ang III and by local applications of Ang II into the SFO, PVN, lateral septal area (LSA), bed nucleus of the stria terminalis (BNST), RVLM, and NTS [139•, 140, 141, 142••]. The central pressor action of Ang II is mediated by the sympathoadrenal system and by AVP acting on V1 receptors [137•, 142••, 143, 144, 145•]. At the cellular level, the central pressor effect of Ang II is mediated by AT1R and is associated with activation of the Rho/Rho-kinase pathway, increased production of O^*-^generated by NADPH oxidase, enhanced stimulation of P38 MAPK and ERK1/2, and decreased SK current in the PVN neurons projecting to the RVLM [146–148]. Local application of Ang II decreases the frequency of GABAergic miniature postsynaptic inhibitory currents in the PVN and it is likely that Ang II excites PVN neurons projecting to the brain stem and spinal cord via an inhibition of GABAergic input to the PVN. As the inhibitory effect of Ang II could be abolished by pertussis toxin and superoxide dismutase, it has been postulated that the inhibition is mediated by the pertussis toxin-sensitive G(i/o) protein and superoxide anions [149]. Acting in the PVN, Ang II potentiates also the cardiac sympathetic afferent reflex [150]. It is likely that sympathoexcitation induced by stimulation of AT1R may be partly reduced by parallel stimulation of AT2R as it has been shown that ICV infusion of Compound 21, a non-peptide AT2R agonist, decreases norepinephrine secretion and blood pressure via the NO signaling pathway in the PVN [151]. Moreover, ICV Ang II injections in AT2R gene knockout mice resulted in higher blood pressure elevations than in wild-type mice [152]. Resetting of AT1R and AT2R activation has been demonstrated in rats with chronic heart failure (CHF) as they manifest significantly higher pressor, tachycardic and sympathoexcitatory responses to stimulation of AT1R in the RVLM. They also show markedly reduced hypotensive, bradycardic, and sympathoinhibitory responses to activation of RVLM AT2R than the sham-operated individuals [153].

#### Regulation of the Autonomic Nervous System and Cardiovascular Reflexes by Angiotensins

Numerous studies have provided evidence that Ang II is an essential modulator of the transmission of signals in the sympathetic and parasympathetic portions of the autonomic nervous system (ANS) and that local changes of Ang II concentration in the ganglia may play an essential role in the final adjustment of the ANS activity to specific physiological/pathological conditions. Dysregulation of the cardiovascular system by the ANS with enhanced activity of the sympathetic nervous system (SNS) and reduced parasympathetic nervous system (PNS) tone is among the characteristic symptoms of hypertension and cardiac failure [154, 155].

At the supraspinal level, Ang II stimulates the presympathetic neurons of the PVN and pacemaker cells of the RVLM. It also depolarizes the preoptic neurons and increases resting K^+^ conductance in the bulbospinal neurons of the C1 area. There is a positive feedback loop between Ang II and SNS, i.e., Ang II stimulates the presympathetic neurons of the PVN and RVLM and facilitates sympathoadrenal transmission [156], whereas sympathetic stimulation enhances the release of renin in the kidney [157]. Circulating Ang II may increase presympathetic stimulation in the central nervous system (CNS) through binding to AT1R in certain autonomic regions, which have no blood-brain barrier, such as the area postrema (AP) and SFO [158, 159].

Numerous studies have provided evidence for the involvement of Ang II and AT1R in the regulation of the peripheral sympathetic system [160]. Angiotensin-immunoreactive neurons are present in the sympathetic ganglia and Ang II facilitates the transmission of signals from the preganglionic to the postganglionic neurons, enhances the release of norepinephrine (NE) during ganglionic excitation, and promotes the release of NE from the sympathetic termini [161, 162]. Abundant specific AT1 receptor binding sites were found at the somatodendritic membrane of the superior cervical ganglion neurons [163]. In the aortic, renal, and celiac ganglia, Ang II was able to trigger an increase in [Ca^2+^]_i_ through an effect dependent on the inflow of extracellular calcium via specific N-type and L-type calcium channels and activation of PKC [160, 164, 165]. Acting on AT1R in the superior cervical ganglion, Ang II modulates also M type K^+^ channels and intensifies the I_Kv_ current through an ATP-dependent effect, which is mediated by PKC [165, 166].

Ang II receptor binding sites have been found along the vagal components subserving the baroreceptor reflex, including the nodose ganglion, NTS, and the dorsal motor nucleus of the vagus (DVMNc) [167]. Impaired efferent vagal tone was found in rats with myocardial infarction and the impairment could be attenuated by the administration of losartan [168]. Reduced parasympathetic tone is also found in human patients with chronic heart failure and can be improved by ACE inhibitor therapy [169, 170]. AT1 receptors are expressed at multiple sites of the baroreceptor reflex pathway including the sensory afferent neurons and their terminals in the NTS [171•]. It has been shown that denervation of baroreceptors significantly increases pressor responses to the central application of Ang II, which argues for an essential role of baroreceptors in buffering the central pressor effect of this peptide [172]. However, during administration of Ang II, the buffering role of baroreceptors is not as effective as in the case of other vasoconstrictory substances and this is related to the resetting of the baroreflex by Ang II. Namely, during administration of Ang II, the baroreflex relationship between blood pressure and heart rate was shifted to a higher pressure level [173]. Ang II tonically modulates cardiovascular regulation through an effect on neurons forming the baroreflex circuitry in the NTS, RVLM, and caudal ventrolateral medulla (CVLM) [174•, 175–177]. Enhanced stimulation of AT1R in the CVLM is presumably responsible for the inhibition of the cardiac vagal baroreflex gain during neurogenic stress [178], whereas AT1R located in the NTS may participate in the transmission of exaggerated excitation from the cardiac sympathetic afferents and in the potentiation of the chemoreceptor reflex during hypoxia [179]. There is also evidence that overactivation of central AT1R accounts for the desensitization of the baroreflex function, elevated sympathoexcitation, and decreased sympathetic inhibition after myocardial infarction in rats and rabbits [180, 181].

In contrast, stimulation of AT2R potentiates baroreceptor reflex sensitivity, though this effect is normally masked by the stimulation of AT1R [182]. Inhibition of ACE 2 in the NTS significantly reduces the reflex heart rate control [183], whereas microinjections of Ang-(1-7) and the transfer of the ACE 2 gene into the NTS of the SHR, as well as microinjections of Ang-(1-7) into the CVLM of rats with renovascular hypertension significantly improve the baroreflex control of the heart rate [184, 185]. On the other hand, administration of Ang-(1-7) into the RVLM of rats with renovascular hypertension appears to exert the opposite effect, as it has been found that it enhances the cardiac sympathetic afferent reflex and increases the sympathetic outflow and blood pressure via Mas receptor activation and stimulation of the cAMP-protein kinase A (PKA) pathway [186].

Interestingly, in the majority of sensory neurons of the nodose ganglion, Ang II inhibited [Ca ^2+^]_i_ in conotoxin-sensitive calcium channels. However, the facilitation of the calcium current by Ang II, involving activation of a reserve pool of dihydropyridine sensitive channels, could be also observed after blockade of conotoxin-sensitive channels [187].

#### Interaction of Ang II with Vasopressin and Aldosterone

The presence of the (pro)renin receptor, AT1R and AT2R mRNAs in the PVN and supraoptic nucleus (SON) and their co-localization with AVP in the magnocellular neurons strongly suggests that the RAS plays a significant role in the regulation of the brain vasopressinergic system [188]. Indeed, several studies have shown that ICV infusion or local application of various constituents of the RAS into the PVN and SON elicits the release of AVP [105, 189, 190]. There is also evidence that overactivation of the RAS may play a significant role in the increased release of AVP in cardiovascular pathology. For example, blockade of AT1R or ACE was found to enhance central and peripheral release of AVP in experimental left ventricular hypertrophy induced by aortic banding. Moreover, experiments on transgenic rats (TG) carrying the mouse renin gene showed a significantly greater content of AVP in the dorsal lower brain stem and an elevated intrabrain release of AVP in response to Ang II application [105]. Angiotensin II plays a significant role in the regulation of the synthesis and release of aldosterone from the adrenal gland [191]. Moreover, there is cross-talk between AT1Rs and mineralocorticoid receptors (MR) in the cardiovascular system so that the stimulation of AT1R increases the effectiveness of the action of aldosterone [192]. Mineralocorticoids increase the expression of Ang II receptors in the arterial smooth muscle cells whereas blockade of AT1R reduced the pro-oxidant effect of aldosterone on the arterial wall [193, 194]. In VSMC, aldosterone potentiated Ang II-induced ERK-1/2 and JNK phosphorylation and increased NADPH oxidase activity. The latter effect was particularly prominent in the mouse model of atherosclerosis [195].

#### The Renin-Angiotensin System and the Water-Electrolyte Balance

There is substantial evidence that various components of the RAS, and especially Ang II, considerably contribute to the regulation of fluid balance through parallel stimulation of sodium and water intake and sodium and water retention. Central or peripheral infusions of renin or angiotensins stimulate sodium ingestion [196, 197]. The circulating Ang II stimulates salt appetite by an action exerted at the forebrain circumventricular organs (CVO) [198]. The stimulation is mediated by AT1R and AT2R and is potentiated by aldosterone [197, 199].

Renin, Ang II, and Ang III are potent dipsogens and avid stimulation of thirst belongs to the most spectacular effects of these peptides. Several studies indicate that the dipsogenic action of Ang II engages AT1R in the median preoptic nucleus and lateral septal area [200–203]. The dipsogenic effect is adjusted to the actual blood volume and hemodynamic conditions and can be partly damped by the parallel inhibition of thirst by an enhanced input from baroreceptors and cardiopulmonary receptors. These mechanisms are partly abolished in hypertension and heart failure [197, 202, 204].

Angiotensin AT1Rs are widely distributed in the kidney [205]. As discussed in several studies, stimulation of the RAS causes retention of sodium and water and this results both from the direct renal effects of Ang II and from the stimulation of aldosterone secretion [206–208]. Acting directly in the kidney via AT1R, Ang II constricts the smooth muscle cells of afferent and efferent arterioles and increases sodium absorption in the proximal tubule. This is associated with a reduction of the renal cortical and papillary blood flows, impairment of tubuloglomerular feedback sensitivity, and resetting of the pressure natriuresis relationship [44, 209]. Removal of one or several of the genes of the RAS results in significant disturbances of the renal functions, manifested by polyuria and inefficient urine concentration, and in abnormalities of the renal structure [210, 211]. Overactivation of the RAS associated with increased activity of aldosterone, vasopressin, and TGF-β are considered to be key pathogenic factors in chronic kidney diseases [206, 212•].

## Vasopressin

### Organization of the Vasopressinergic System

Arginine vasopressin, the principal vasopressin peptide in mammals, is synthesized mainly in the neuroendocrine neurons of the supraoptic, paraventricular, and suprachiasmatic nuclei of the hypothalamus. Vasopressin expressing cells have also been identified in the BNST, medial amygdaloid nucleus, dorsomedial hypothalamic nucleus (DMH), nucleus of the diagonal band of Broca (DBB), and the olfactory bulb [213–215]. The magnocellular neurons (MCN) of the SON and the PVN send axons to the capillary vessels of the posterior pituitary where they release their products into the systemic circulation. Circulating AVP is involved in the regulation of the water-electrolyte balance, blood pressure, and metabolism. Some of the neurons send axons to the median eminence and release AVP into the hypophyseal portal system and form a link regulating the secretion of ACTH and other pituitary hormones. The parvocellular neurons of the PVN, DBB, BNST, and suprachiasmatic nucleus (SCN) send axons to multiple regions of the CNS located at virtually all levels of the brain, including the brain cortex, brain stem, CVO, and the spinal cord [216, 217, 218•, 219]. The CNS vasopressinergic neurons participate in the regulation of cardiorespiratory functions, the water-electrolyte balance, circadian rhythmicity, food intake, metabolism, pain, neurogenic stress, emotions, social behavior, and immunological processes. There is evidence for a dendritic release of AVP and its role in the regulation of local neuronal activity [220]. Some evidence indicates that synthesis of AVP occurs in the heart, adrenal medulla, and pancreas [221–223].

#### Regulation of the Release of Vasopressin. Role of Angiotensins and Mineralocorticoids

The release of vasopressin is significantly influenced by changes in plasma osmolality, blood volume, and blood pressure. At the cellular level, osmotic stimulation of AVP release involves the activation of transient receptor potential cation channel subfamily V member (TRPV1 and TRPV4) channels [224, 225]. Hypothalamic AVP synthesizing neurons receive inputs from multiple neurotransmitting and neuromodulating neurons releasing catecholamines, serotonin, acetylcholine, histamine, angiotensins, neuropeptide Y, orexins, substance P, glutamate, and ATP. Several cardiovascular compounds, including Ang II, have receptors in the PVN and SON and stimulate the release of AVP [212•, 226–228]. The inhibition of AVP release occurs after administration of GABA, NO, natriuretic peptides, apelin, and relaxin-3 [229–232]. As discussed above, Ang II is a potent stimulator of AVP release. In the context of angiotensinergic-vasopressinergic interactions, special attention should be given to the role of GABA, serotonin, and steroid hormones. In most studies performed on normotensive animals, stimulation of the GABAA receptors resulted in the inhibition of vasopressinergic neurons and was mediated by Cl^−^ influx via Na^+^,K^+^,2Cl^−^ cotransporter (NKCC1) channels [233]; however, an excitatory effect of GABAA on AVP secreting neurons was also observed [234] and it has been suggested that the inhibitory input of GABA innervation may be replaced by an excitatory drive under some pathological circumstances, for example, in DOCA-salt hypertension [235]. At present, it seems that the release of AVP can be adjusted to specific conditions by means of different neuroactive modulators. For example, different types of serotonin (5-HT2A, 5-HT2C, 5-HT3, and 5-HT4), Ang II, and cytokine receptors are engaged in vasopressin release during various types of stress [30, 212•, 236].

The role of steroid hormones in the regulation of AVP secretion has been explored in detail. Specific 11β-HSD2 immunoreactivity and mineralocorticoid receptors were identified in the cytoplasm of the SON and PVN neurons [237]. Colocalization of MR with the membrane conductance regulating epithelial sodium channels (ENaC) was found in the MCN neurons immunoreactive for AVP. Interestingly, both normotensive and SHR rats respond with the upregulation of AVP mRNA and V1a receptors to the administration of mineralocorticoids, however, SHR rats show greater expression of AVP, V1aR, and MR than WKY rats [238•]. Moreover, abundant expression of MR and α, β, and γ subunits of ENaC are present in the cardiovascular brain regions innervated by vasopressinergic neurons [239, 240].

### Cellular Effects of Vasopressin

Arginine vasopressin acts via three primary types of receptors (V1aR, V1bR, and V2R) which belong to the family of monomeric G protein receptors (GPCR) [241, 242].

#### V1 Receptors

Vasopressin V1aR have been detected in the brain, heart, vessels, lungs, kidney, liver, pituitary gland, adrenals, uterus, and in other peripheral organs. In the nervous system, V1aR mRNA and protein were identified at virtually all levels of the brain and spinal cord [212•, 243•, 244•, 245•]. There is also functional evidence for the presence of V1aR in the glial cells of the brain and dorsal ganglia. These cells responded to the application of AVP with an increase of intracellular Ca^2+^ concentration, which could be abolished by V1aR and PLC antagonists [246]. In the heart, V1aR were detected in the cardiac muscle and in the coronary vessels. In the kidney, they were found in vessels (interlobular arteries, efferent arterioles, vasa recta, glomerular mesangium) and in the JGA, the macula densa, thick ascending limb of the loop of Henle, the distal convoluted tubule, and the principal and intercalated cells of the collecting ducts [247, 248, 249•].

Distribution of V1bR is less widespread than of V1aR; however, they have been detected in the brain, the pituitary gland, and the pancreas [212•, 250•, 251–252].

Stimulation of V1R activates intracellular pathways engaging Gq/11/PLC/IP3/PKC/MEK1/2/MAPKK, PI3-kinase, calcium-calmodulin kinase, and adenylyl cyclase of type III. The receptors are coupled to phospholipase C via a G protein of the Gq/11 type and their activation results in generation of inositol 1,4,5-triphosphate (IP3). In smooth muscle cells, IP3 interacts with IP3 receptors (IP3R) of the sarcoplasmic reticulum (SR), and the subsequent release of calcium results in stimulation of the ryanodine receptors (RyR). In cells expressing adenylyl cyclase of type III, stimulation of V1R may also potentiate cAMP accumulation, induced primarily by exposure to isoproterenol or prostaglandin I_2_ (PGI_2_) [253•]. More recently, it has been shown that AVP significantly modulates the ionic transport of vascular smooth muscle cells. In picomolar concentrations, it elicits calcium transients that are associated with activation of protein kinase C (PKC) and the inflow of calcium through voltage-sensitive L-type channels (VSCC) [254•, 255•, 256•]. In A7r5 vascular smooth muscle cells, application of 100 pm of AVP increases the frequency of calcium spikes through an effect associated with proline-rich tyrosine kinase 2 (PYK2), tyrosine kinase phosphorylation and activation of PKC. Vasopressin also regulates the potassium current engaging the delayed rectifier K^+^ channels, and this effect depends on phosphorylation of the Kv1.2 channel protein and inhibition of the voltage-dependent potassium current (I_Kv_), which is associated with gradual membrane depolarization and the generation of action potentials [256•]. In A-10 rat aortic smooth muscle cells, AVP stimulates sodium-dependent phosphate transport through a mechanism involving activation of V1R and multiple V1R-dependent signaling pathways, such as PKC, PI3-kinase, S6 kinase, and Jun kinase [257•].

In some forms of hypertension, there is an overactivation of the vasopressinergic system. A higher number of AVP mRNA expressing cells and V1aR-immunopositive cells has been found in the PVN of the SHR than in the PVN of WKY rats. In the SHR, both these parameters were significantly elevated after the administration of DOCA, whereas WKY rats responded only with an increase in the number of V1aR-positive cells [238•]. On the other hand, a decreased expression of V1aR mRNA in the brain medulla and an increased expression of V1bR mRNA in the mesencephalon-pontine region were found in hypertensive renin transgenic TGR(mRen2)27 rats in comparison with the parent normotensive SD strain. The TGR(mRen2)27 rats manifested also a higher expression of V1aR in the renal medulla than SD rats [244•, 250•]. Significant changes in expression of V1aR in the brain and kidneys were also found in rats with renovascular hypertension, in post-infarct heart failure, and in rats exposed to chronic stress [258•, 259••].

#### V2 Receptors

Vasopressin V2R are expressed mainly in the kidney where they are located on the basolateral membrane of the thick ascending limb (TAL), the distal convoluted tubule, and the collecting ducts [249•, 260•]. The stimulation of V2R causes activation of adenylyl cyclase and formation of cAMP. This is followed by an activation of protein kinase A, and a subsequent phosphorylation of aquaporin 2 (AQP2). The phosphorylated AQP2 is subsequently incorporated into the luminal membrane of the cell and serves as a water channel. Vasopressin also regulates the transcription of the AQP2 gene [261•, 262•, 263•].

#### Dimers and Dual Receptors

Studies on cell cultures have shown that AVP receptors can form various variants of homo- (V1aR/V1aR, V1bR/V1bR, V2R/V2R) and heterodimers (OTR/V1R, V1aR/V2R, V2R/OTR, V1bR/CRH). It is likely that dimerization plays a role in the process of intracellular internalization of the receptors and may determine the effectiveness of the cellular action of AVP. Thus far, there is no evidence for the dimerization of vasopressin and angiotensin receptors. This issue was approached in the study of Szalai et al. [264] who were unable to prove the formation of heterodimers of vasopressin V2R with angiotensin AT1R or AT2R.

A dual Ang II/AVP receptor coupled to adenylate cyclase has also been characterized and its immunocytochemical distribution in the kidney has been determined. The immunopharmacological characteristics of this receptor indicated that it may have functional properties of the AT1R and V2R. It has been suggested that it may play an essential role in sodium and fluid resorption in the inner medullary collecting ducts [265•].

### Role of Vasopressin in Cardiovascular Regulation

It has long been known that moderate elevation of AVP in the systemic circulation increases arterial blood pressure, central venous pressure, and central blood volume, and resets the reflex regulation of blood pressure by baroreceptors [266•, 267•]. Similarly as Ang II, AVP participates in the regulation of blood pressure via multiple actions exerted either directly on vascular smooth muscle cells and cardiac myocytes or indirectly through the regulation of the water-electrolyte balance and blood volume. Besides, AVP stimulates the pre-autonomic neurons in the brain as well as modulates the synaptic transmission in the sympathetic ganglia [268•].

#### Direct Vascular Effects of Vasopressin

Vasopressin is one of the most potent vasoconstrictors, although responsiveness of various vascular beds to its contractile action significantly differs. The most sensitive vessels to vasopressin are those of the skin, muscle, and splanchnic circulation [269•, 270•]. The vasoconstrictory effect of AVP is mediated by V1aR. Vasopressin elicits contractile and growth-promoting effects in vascular smooth muscle cells, increases smooth muscle actin and SM22 promoter activities, and stimulates the JNK and p38 MAPK pathways [271•]. Moreover, experiments on ring segments of gastroepiploic arteries obtained from human patients have shown that AVP is able to restore the contractile response to Ang II after tachyphylaxis [270•]. The vasoconstrictory potency of AVP may be significantly altered under pathological conditions [272•].

The interaction of Ang II, AVP, and aldosterone in the regulation of cellular sodium content in the rat tail arteries was approached by Friedman [273•], who showed that exposures to aldosterone, Ang II, and AVP were associated with a significant reduction of free cell Na^+^ and that the effects of Ang II and AVP were additive with the effect of aldosterone.

#### Direct Cardiac Effects of Vasopressin

Vasopressin stimulates IP3 accumulation in the atrial and ventricular cardiomyocytes [274•]. Current evidence indicates that V1aR is the main type of AVP receptors in the adult heart although it appears that, in newborns, V2R are also present and may play an essential role in differentiation of the embryonic stem cells into contracting cells which display cardiac specific transcription factor GATA-4 and ventricle-specific myosin light chain [275•]. Studies in vivo have shown that transfer of the V2R gene into the myocardium increases cardiac contractility [276•]. It has been suggested that by affecting cardiac contractility, AVP may contribute to adjustment of newborns to the hypoxic conditions during posthypoxic reoxygenation [277•].

There is evidence that excessive stimulation of V1aR promotes pathological changes in the heart. It has been shown that it causes cardiac hypertrophy and fibrosis through increased synthesis of collagen and TGF-β1, and enhanced proliferation and differentiation of fibroblasts. Exposure of the cultured neonatal cardiomyocytes for 24 h to AVP resulted in an increase of the cell surface areas, elevation of expression of the atrial natriuretic peptide (ANP), and activation of ERK1/2 via the V1aR-dependent effect. It was also shown that V1aR-deficient mice did not respond with cardiac hypertrophy to pressure overload [278•]. The role of AVP in the regulation of the cardiac functions partly depends on its interaction with NO. Vasopressin increases inducible nitric oxide synthase (iNOS) expression and NO production, and activates NF-κB. Moreover, blockade of NO synthase partly eliminated the effects of i.v. infusion of AVP in adult pigs [279•]. The profibrotic effect of AVP in the heart of the adult pig can be inhibited by stimulation of Mas receptors by Ang-(1-7). In AVP-stimulated cardiac fibroblasts, Ang-(1-7) was found to reduce activity of calcineurin and to inhibit nuclear translocation of NF-κB and its effect on transcriptional activity [280•, 281•].

Experimental and clinical studies have provided evidence that blood AVP concentration is significantly elevated in hypertension and in patients with post-infarct cardiac failure [282••, 283•]. Moreover, experimental studies revealed that, in post-myocardial infarction, cardiac dysfunction is associated with increased cardiac and plasma AVP and Ang II expressions, and that AVP expression is positively correlated with left ventricular end diastolic diameter (LVEDD) [284]. The same authors have shown that, under in vitro conditions, cardiac microvascular endothelial cells respond with increased expression of AVP mRNA and protein to the application of Ang II. It has also been shown that AVP promotes proliferation of those cells. Prolonged exposure of neonatal rat cardiac fibroblasts to elevated AVP levels promoted their proliferation and induced increased expression of matrix metalloproteinases (MMP2 and MMP9). These effects were mediated by the stimulation of V1aR and activation of G protein-coupled receptor kinase 2 (GRK2) that is followed by phosphorylation of ERK1/2 [285].

Effects of prolonged exposure of the heart to stimulation of V1aR were explored in the study of Li X et al. [286••], who found that mice with cardiac-targeted overexpression of V1aR develop cardiac hypertrophy associated with reduced cardiac contractility and dysfunction of isolated cardiomyocytes. The latter effect was manifested by reduced amplitude and elongated duration of action potentials of the myocytes, and this was associated with a reduction in [Ca2^+^]_i_. Insulin-mediated ERK1/2 phosphorylation was enhanced whereas Akt-induced and JNK-induced phosphorylations were not altered. The cardiac effects of overexpression of V1aR were reduced by inhibition of the Gαq/11 transduction pathway [286••].

#### Direct Renal Effects of Vasopressin

Stimulation of V1aR by AVP is involved in the regulation of glomerular filtration and renal circulation. Acting on V1aR, AVP enhances the calcium influx into the smooth muscle cells of the preglomerular arterioles, constricts the afferent and efferent arterioles, and contracts the mesangial cells [287•, 288•, 289•]. In the glomerular mesangial cells of the cultured rat, AVP stimulated mitosis and growth of the cells and increased the intracellular calcium content. The mitotic response to AVP was stimulated by insulin. Acidification followed by alkalinization was shown after exposure of the mesangial cells either to AVP or to Ang II and the alkalinization depended on the activation of the Na^+^,H^+^ exchange [290•, 291•]. It has been proven that the inner medullary region of the kidney circulation is more sensitive to V1aR stimulation than the cortical vessels and that AVP can regulate the renal papillary blood flow in physiological concentrations [292•, 293•, 294•].

#### Central Cardiovascular Effects of Vasopressin

Centrally projecting vasopressinergic neurons innervate several groups of neurons engaged in cardiovascular control such as the NTS, RVLM, DVMNc, PBN, amygdala, BNST, periaqueductal gray matter, striatum, and the prefrontal cortex [212•, 218•, 219•, 295•]. There is significant evidence that centrally acting AVP may evoke both pressor and hypotensive responses [137•, 145•, 296•, 297•] and that the final effect depends on the stimulation of specific groups of vasopressinergic fibers innervating special sets of cardiovascular neurons and activated by specific stimuli.

#### Vasopressin in the Regulation of the Autonomic Nervous System and Cardiovascular Reflexes

Similarly as the RAS, the vasopressinergic system closely cooperates with the autonomic nervous system by means of multifarious interactions. In the brain, the presympathetic neurons of PVN and RVLM are innervated by vasopressinergic fibers and are furnished with V1a receptors. Moreover, the vasopressinergic fibers project to the spinal cord [212•, 218•, 219•, 243•, 245•]. AVP-expressing neurons furnished with synaptic-like contacts were also identified in the dorsal vagal complex, specifically in the DVMNc, and in the NTS subnuclei. In the A2/C2 and A2 catecholaminergic neurons and in the nucleus ambiguus, AVP positive fibers make contacts with tyrosine hydroxylase (TH) positive neurons [295•]. Vasopressinergic neurons and V1R are present in the RVLM and the rostral ventral respiratory column (RVLC) and expression of V1aR in this region increases during hypoxia whereas blockade of V1aR there reduces pressor responses to hypoxia [298••].

Studies using viral transneuronal labeling and immunohistochemistry revealed that the PVN is the source of vasopressinergic and angiotensinergic neurons projecting to the stellate ganglion, which is the main source of sympathetic innervation of the heart [299••]. Furthermore, experiments on sympathoadrenal preganglionic neurons have provided evidence that some of the PVN neurons have terminal varicosities closely associated with SPNs and that vasopressin fibers are likely to form synaptic contacts with SPN [268•]. Vasopressin-like peptide and V1R were identified in the sympathetic ganglia, and application of AVP has been shown to stimulate inositol lipid breakdown in the ganglia [300••, 301••]. The functional significance of the presence of AVP in the peripheral autonomic nervous system is not yet fully clear. Iontophoresis of AVP on antidromically identified sympathetic preganglionic neurons in the second thoracic (T) segment of the spinal cord results in the inhibition of the majority of neurons, however, some of the neurons are excited [302••]. ICV administration of AVP causes c-fos induction in adrenergic and noradrenergic cells of the adrenal medulla, which may argue that centrally released vasopressin has an impact on the activity of the preganglionic sympathetic neurons innervating the catecholaminergic neurons of the adrenal glands [303••]. Peripheral administration of AVP significantly potentiated the vasoconstrictory effect of lumbar sympathetic nerve stimulation and of norepinephrine administered into the isolated perfused hind limb of the rabbit through an effect mediated by V1R [304••].

Several studies have provided evidence that AVP plays an essential role in the potentiation of the baroreflex, although there are interspecies differences in magnitude of this effect [266•, 305••, 306••, 307••]. Most of the studies have shown that AVP facilitates the baroreflex by means of V1aR located in the area postrema and the NTS [308••, 309••, 310••].

#### Vasopressin and the Water-Electrolyte Balance

Vasopressin participates in the regulation of the water-electrolyte balance through the regulation of water and electrolyte transport in the renal tubules and through the control of sodium and water intake. These effects are exerted either by means of its independent action or through cross-talks with Ang II and aldosterone.

Reabsorption of water in the collecting ducts requires activation of V2R and is the most prominent effect of AVP in the kidney. The effectiveness of this process is determined by the availability of AVP and active V2R; however, it also depends on the effectiveness of the transport of electrolytes and the regulation of renal blood flow. In the collecting ducts, AVP binds to V2R on the basolateral membrane of the principal cells and this results in the immediate activation of adenylyl cyclase and formation of cAMP. Subsequent stimulation of PKA results in the phosphorylation of AVP-sensitive water channels (AQP2) and their translocation to the apical tubular membrane. Apical insertion of AQP2 is coupled to calcium mobilization from ryanodine-gated intracellular calcium stores and to extracellular Ca^2+^ import via the capacitative calcium influx [311••]. Prolonged AVP-induced intracellular cAMP elevation also increases AQP2 gene transcription and AQP2 abundance [312••].

In the principal cells of the collecting ducts (mpkCCD, c14 cell model), AVP stimulates transepithelial Na^+^ transport and increases sodium absorption by means of active NKA and passive Na^+^,K^+^,2Cl^−^ (NKCC2) transports. Activity of NKA is constitutively regulated by the ERK1/2 pathway (p42 and p44 mitogen-activated protein kinase pathways). It is suggested that the basal activity of the ERK1/2 pathway plays a permissive role in the activation of Na,K-ATPase. Vasopressin, like Ang II and aldosterone, induces the translocation of NKA from the cytoplasm to the plasma membrane and facilitates its activation [313••, 314••]. In the thick ascending limb of the loop of Henle, AVP enhances passive sodium transport by means of NKCC2 cotransport [315••]. In the collecting ducts, it facilitates permeability to urea and plays an essential role in the regulation of the epithelial sodium transport mediated by ENaC. The latter effect involves interaction of AVP with aldosterone [316•, 317•].

#### Interaction of AVP with Aldosterone

Vasopressin stimulates the release of aldosterone from the dispersed adrenal gland cells through an effect mediated by V1aR [318•]. The presence of AVP is also necessary for the effective action of aldosterone in the renal tubules. Acting on V2R in the collecting ducts, AVP increases the catalytic activity of 11β-HSD2, which causes cortisol to be converted into inactive cortisone. As a result, MR receptors can be stimulated more selectively by aldosterone [319•]. AVP is also necessary for full activation of the ENaC channels by aldosterone in the distal part of the nephron. The excessive activation of these channels during prolonged stimulation of MR results in sodium retention and the elevation of blood pressure while the inhibition of action of aldosterone causes a decrease in ENaC activity and sodium loss [320•]. There is evidence that, in some instances, AVP can partly compensate the inefficient action of aldosterone in the collecting ducts. This is particularly essential in adrenocortical insufficiency, during which the nonosmotic stimulation of AVP release is elevated because of body fluid depletion and the lack of inhibition of AVP release by glucocorticoids [273•, 321•]. Experiments on adrenalectomized mice, with an aldosterone concentration below the lower limit of quantification, revealed that those mice manifest a thriving expression of the ENaC channels, and that those channels can be stimulated by AVP acting on V2 receptors [322•, 323•]. Experiments on cells of the cortical collecting duct (CCD) have shown that AVP and aldosterone act synergistically to promote sodium reabsorption acting both at the apical and the basolateral membranes. In the epithelial membrane, AVP is necessary for full sensitivity of ENaC to aldosterone while, in the basolateral membrane, aldosterone and AVP cooperatively recruit NKA from the intracellular stores and promote their translocation to the membrane. It has been shown that aldosterone produces delayed increases of mRNAs encoding alpha subunits of the ENaC (sixfold), and of alpha1 subunits of NKA, whereas AVP induces rapid translocation of NKA into the basolateral membrane [324•]. It has been shown that vasopressin, similar to aldosterone, promotes the synthesis of α- and γ-ENaC subunits [325•].

Experiments using molecular engineering indicate that V1aRs are engaged in the regulation of the acid-base balance via aldosterone. Thus, mice deprived of V1aR developed type 4 renal tubular acidosis and manifested a reduced expression of MR and 11β-HSD2 in the kidney medulla. In addition, transgenic rats expressing MR but deprived of V2R did not respond with an increased expression of H^+^,K^+^-ATPase, H^+^-ATPase, and C glycoprotein of the Rh family to the administration of aldosterone after an additional knockdown of the V1aR gene. Moreover, it was shown that, in V1aR(+/+) mice, AVP is able to increase the expressions of protein kinase C alpha (PKCα) and protein kinase C beta1 (PKCβ1), whereas aldosterone elevates the expressions of protein kinase C delta (PKCδ) and protein kinase C zeta (PKC-ζ). These effects are abolished by the knockdown of V1aR. These studies indicate that, in the renal tubules, V1aR cooperates with aldosterone in a complex manner, which has a significant impact on sodium reabsorption and may include regulation of MR translocation from the cytoplasm to the nucleus [326•, 327•].

## Significance of Central and Peripheral Interactions of the RAS and the VPS

**Fig. 1:**
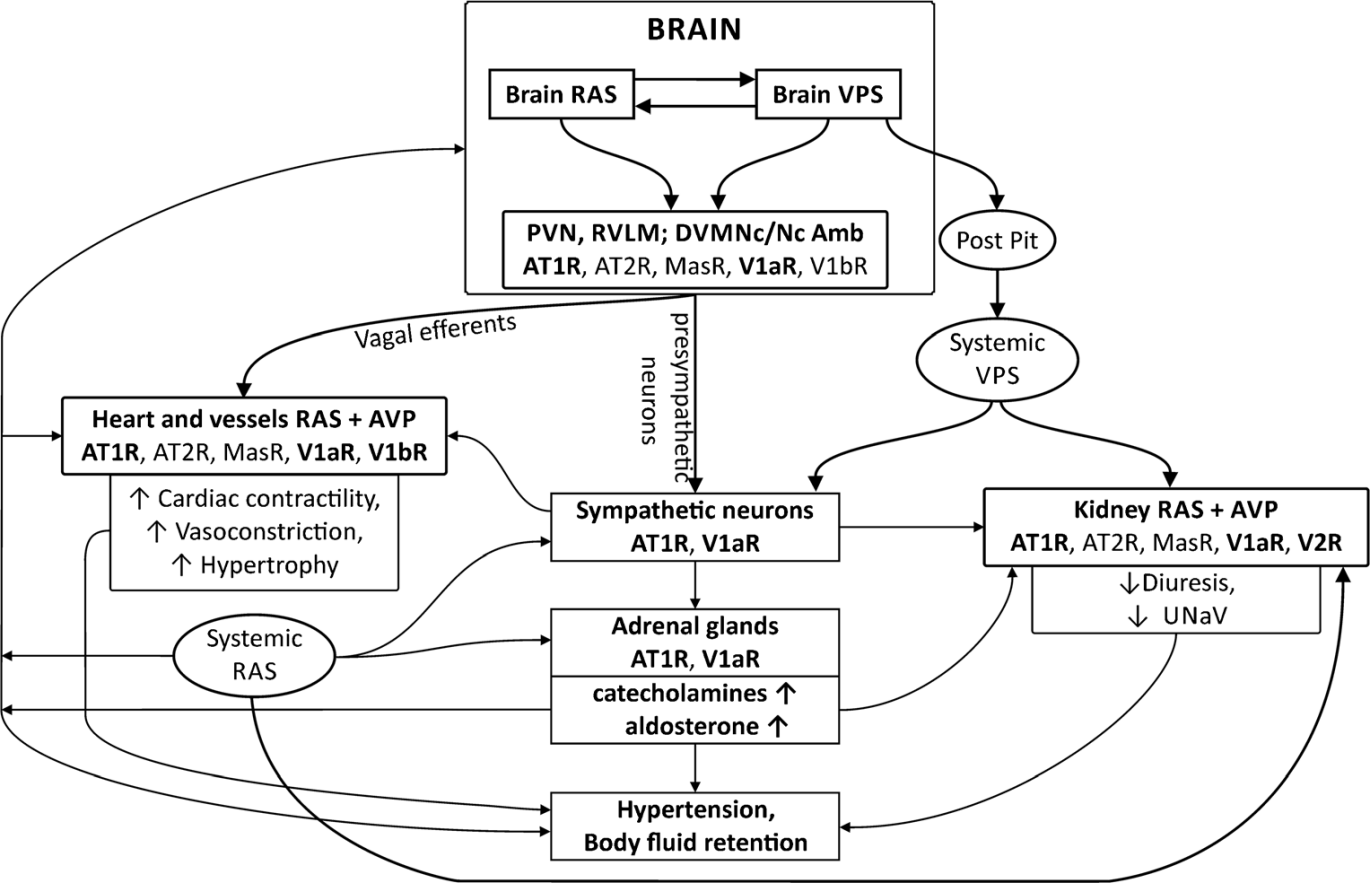
Interactions of the renin-angiotensin system (RAS) with the vasopressinergic system (VPS) in the regulation of blood pressure and body fluid volume. RAS and VPS closely cooperate in adjusting blood pressure to cardiovascular challenges. The cooperation takes place in the cardiovascular regions of the brain, in the cardiovascular and the sympathoadrenal systems, and in the kidney. Multiple synergistic and/or antagonistic actions of angiotensin peptides and vasopressin, as well as positive and negative feedbacks between RAS and VPS are involved in the regulation of cardiovascular functions. The figure shows that dysregulated interaction of RAS and VPS in the brain and in the peripheral tissues results in excessive stimulation of angiotensin AT1 receptors (AT1R), and vasopressin V1a (V1aR) and V2 (V2R) receptors, and in the development of hypertension and/or body fluid retention. AT2R, angiotensin AT2 receptors; AVP, arginine vasopressin; DVMNc/Nc Amb, complex of the dorsoventromedial nucleus of the vagus and the nucleus ambiguous; MasR, Mas receptor of angiotensin- (1-7); Post Pit, the posterior pituitary; PVN, the paraventricular nucleus of the hypothalamus; RVLM, the rostral ventrolateral medulla of the brain; UNaV, sodium excretion

The above survey indicates that, in many instances, there are multiple interactions of angiotensin II and vasopressin and that the close cooperation of these two peptides is a condition of the fully efficient regulation of blood pressure and body fluid volume. As shown in Fig. 1, Ang II and AVP interact reciprocally at several levels through (1) mutual regulation of their own release, (2) targeting the same groups of cells, and (3) engaging the same intracellular pathways.

### Interactive Regulation of the RAS and the VPS

Angiotensin II is a potent stimulator of AVP release. The mechanism underlying this effect has been partly clarified as it has been shown that, in the PVN, Ang II interacts with nonselective cation channel TRPV4. Application of Ang II to the immortalized neuroendocrine rat hypothalamic 4B cell line (the line expressing transcripts of AVP, AT1R, AT1bR, and TRPV4) significantly elevates the expression of TRPV4 and this effect can be abolished by the blockade of AT1R with losartan. Acting on AT1R, Ang II also potentiated the calcium influx evoked by a selective TRPV4 agonist (GSK 1016790A) [328•]. Thus, it is likely that the release of AVP by Ang II in the PVN neurons involves the activation of TRPV4. In this line, it is interesting to note that rats with liver cirrhosis, manifesting symptoms of the syndrome of inappropriate antidiuretic hormone secretion (SIADH), show overexpression of the TRPV4 channels in the hypothalamic SON neurons immunopositive for AVP [329•].

There is also evidence for the regulation of the RAS by vasopressin. Acting directly on the macula densa cells, AVP stimulates the release of renin and can interact thereby with the RAS in a positive feedback loop. However, this action has a limit, because in high concentrations both Ang II and AVP elevate blood volume and blood pressure, which may in turn result in the stimulation of cardiopulmonary receptors and baroreceptors and in the inhibition of the release of renin and AVP (Fig. 1). The consequences of chronic deficiency of V1aR receptors for RAS were assessed in the study by Aoyagi et al. [330•], which was performed on a model of V1aR−/− mice. The mice manifested reduced concentrations of renin and Ang II in plasma, a decreased expression of renin in the renal granule cells, and lowered expressions of nNOS and COX-2 in the macula densa cells [330•]. The study has provided evidence that the stimulation of V1aR by AVP is necessary to maintain the appropriate release of renin from the granule cells of the kidney and that this action may be associated with altered activities of nNOS and COX-2.

### Cooperative Regulation of the Cardiovascular System by Angiotensins and Vasopressin

Several studies have provided evidence that the joint action of Ang II and AVP is necessary for the appropriate regulation of the vascular tone, cardiac contractility, and the release of other cardiovascular factors (see above and Fig. 1). The cooperative action of Ang II and AVP in the regulation of blood pressure is particularly well observed at the level of the CNS and plays a role both in the maintenance of resting blood pressure and in adjustments to posthemorrhagic hypovolemia, hypoxia, cardiorespiratory disorders, and stress. Several studies have provided evidence that the central pressor action of Ang II can be abolished or significantly reduced by blockade of the brain V1aR and that the combined administration of Ang II and AVP exerts greater changes of blood pressure than those changes observed after the separate application of these peptides. Importantly, the interaction of Ang II and AVP is significantly enhanced in hypertension, post-infarct cardiac failure, and during chronic stress [137•, 145•, 331•, 332•, 333•, 334•]. The elevated release of AVP plays a major role in the development of hypertension in mice with brain-specific hyperactivity of the renin and angiotensinogen genes as it has been shown that the baseline blood pressure can be normalized in this strain by the administration of the nonselective AVP receptor antagonist conivaptan or the V2-selective antagonist tolvaptan [335•]. Electrophysiological studies have shown that AVP and Ang II increase the activity of the same rostrodiencephalic neurons and coactivate neurons in the SFO and organum vasculosum of the lamina terminalis (OVLT) [336•, 337•]. The biochemical background of this interaction is partly clarified by studies showing that Ang II and AVP may jointly regulate calcium transits. Studies on a culture of the area postrema/NTS cells have shown that Ang II and AVP evoke a transient increase in [Ca2^+^] and that this effect can be abolished by pretreatment with specific antagonists of AT1R or V1R [338•, 339•]. In in vivo experiments, blockade of central V1aR effectively abolished the central pressor action of Ang II [137•, 145•].

Although, under most circumstances, Ang II and AVP cooperate synergistically with the sympathetic nervous system, in some instances, they can act antagonistically. The most evident example of such counteraction is the inhibition of the baroreceptor reflex by Ang II and potentiation of this reflex by vasopressin [171•, 174•, 340•]. There is also evidence that Ang II and AVP play different roles in the regulation of respiration during acute hypercapnia. Namely, it has been shown that Ang II acting on AT1R stimulates ventilation and increases the metabolic rate during hypercapnia and that this effect is reduced by the simultaneous stimulation of V1aR by vasopressin [341•].

### Cooperative Regulation of the Water and Electrolyte Balance by Angiotensin and Vasopressin

Deletion of V1aR results in a decrease of renin expression in the JGA cells and in a reduced release of renin and Ang II into the systemic circulation [330•]. There are also interactions between renin, AVP, and Ang II in the renal tubules. Renin is synthesized in the principal cells of the collecting duct (CD) and vasopressin stimulates this process by means of V2R and cAMP/PKA/CREB (cAMP response element-binding protein) pathway [342•]. Furthermore, exposure of the inner medulla collecting duct cells to Ang II increases V2R mRNA in these cells in a dose-dependent manner. This effect is stimulated by PKA and suppressed by PKC [343•]. Blockade of AT1R with losartan was shown to reduce the effect of AVP in the renal tubules [344•]. Specifically, a decrease of AVP-mediated cAMP accumulation in the thick ascending limb of the loop of Henle and restoration of a normal NKCC2 expression was found in rats with myocardial infarction [344•].

Some evidence indicates that chronic stimulation of ATR in the kidney may alter the responsiveness of the renal tubules to vasopressin and that stimulation of AT1R potentiates the effect of dDAVP on AQP2 plasma membrane targeting [345•, 346•]. This effect may play a significant role under some pathological circumstances. For example, rats exposed to severe sodium restriction show elevated concentration of Ang II and symptoms of acute renal failure associated with AVP-resistant urinary concentrating defects. These defects are associated with decreased expressions of AQP1, AQP2, AQP3, Na^+^/H^+^ exchanger, Na^+^,Cl^−^ cotransporter, and Na^+^,K^+^-ATPase. The above abnormalities can be corrected by the administration of a V2 agonist (dDAVP). The authors showed that the beneficial effect of dDAVP crucially depends on cooperation of AT1R with V2R as blockade of AT1R with candesartan completely abolished the dDAVP urine concentrating effect. In a less severe NaCl-restriction rat model, blockade of AT1R increased the fractional excretion of sodium and decreased urine osmolality. It also reduced expression of AQP2 and AQP3 and downregulated phosphorylated AQP2 (p-AQP2) in the inner renal medulla. In vitro experiments performed in the same study revealed that both Ang II and dDAVP increase AQP2 and cAMP expressions [347•].

## Conclusions and Perspectives

Altogether, precise cooperation of Ang II and AVP is necessary for the efficient regulation of blood pressure and body fluid volume. In many instances, the effects of both hormones are significantly modulated by the simultaneous action of Ang-(1-7), mineralocorticosteroids, and other cardiovascular factors. Close cooperation of the RAS and VPS with aldosterone inclines to regard the renin-angiotensin-aldosterone system and the vasopressinergic system as a functional unity. Under physiological conditions, cooperation of Ang II and AVP plays a significant role in the maintenance of cardiovascular homeostasis. Excessive stimulation of the RAS and the VPS may have prolonged negative consequences for the cardiovascular system, resulting from the formation of multiple positive feedbacks between renin, angiotensin II, vasopressin, and aldosterone.

Dysregulation of the interactions between the RAS and vasopressin under pathological conditions may significantly contribute to the generation of disturbances in blood pressure regulation and the development of cardiovascular diseases. A rationalized combined treatment with inhibitors of the RAS system and vasopressin antagonists appears to be a promising tool in the therapy of various forms of hypertension and cardiac failure, especially in patients manifesting evident overactivity of the sympathetic component of the autonomic nervous system or excessive stimulation of mineralocorticoid receptors.
